# Comparative analysis of the growth and biological activity of a respiratory and atheroma isolate of *Chlamydia pneumoniae* reveals strain-dependent differences in inflammatory activity and innate immune evasion

**DOI:** 10.1186/s12866-015-0569-3

**Published:** 2015-10-23

**Authors:** Xianbao He, Yanmei Liang, Michael P. LaValley, Juying Lai, Robin R. Ingalls

**Affiliations:** Department of Medicine, Section of Infectious Diseases, Boston Medical Center, Boston, MA USA; Boston University School of Medicine, Boston, MA USA; Department of Biostatistics, Boston University School of Public Health, Boston, MA USA; Division of Rheumatology, Immunology and Allergy, Brigham & Women’s Hospital, Boston, MA USA

**Keywords:** Chlamydia, Innate immunity, Bacterial pathogenesis, Pneumonia

## Abstract

**Background:**

*Chlamydia pneumoniae* is a common human pathogen that is associated with upper and lower respiratory tract infections. It has also been suggested that *C. pneumoniae* infection can trigger or promote a number of chronic inflammatory conditions, including asthma and atherosclerosis. Several strains of *C. pneumoniae* have been isolated from humans and animals, and sequence data demonstrates marked genetic conservation, leaving unanswered the question as to why chronic inflammatory conditions may occur following some respiratory-acquired infections.

**Methods:**

*C. pneumoniae* strains AR39 and AO3 were used *in vitro* to infect murine bone marrow derived macrophages and L929 fibroblasts, or *in vivo* to infect C57BL/6 mice via the intranasal route.

**Results:**

We undertook a comparative study of a respiratory isolate, AR39, and an atheroma isolate, AO3, to determine if bacterial growth and host responses to infection varied between these two strains. We observed differential growth depending on the host cell type and the growth temperature; however both strains were capable of forming plaques *in vitro*. The host response to the respiratory isolate was found to be more inflammatory both *in vitro*, in terms of inflammatory cytokine induction, and *in vivo*, as measured by clinical response and lung inflammatory markers using a mouse model of respiratory infection.

**Conclusions:**

Our data demonstrates that a subset of *C. pneumoniae* strains is capable of evading host innate immune defenses during the acute respiratory infection. Further studies on the genetic basis for these differences on both the host and pathogen side could enhance our understanding how *C. pneumoniae* contributes to the development chronic inflammation at local and distant sites.

## Background

*Chlamydia pneumoniae* is an obligate intracellular pathogen that infects a wide range of hosts, including mammals, amphibians, and reptiles. The first strain identified was isolated from a child’s conjunctiva during a trachoma vaccine trial in Taiwan in the 1960s, and was designated Taiwan strain TW183 [1]. A second strain was isolated from the respiratory tract in 1983 from the pharynx of a University of Washington student with pharyngitis, and designated acute respiratory isolate AR39. Together they were referred to as the TWAR isolates [2]. The TWAR strains, now known as *Chlamydia* (previously referred to as *Chlamydophila*) *pneumoniae*, are associated with atypical pneumonia as well as pharyngitis, bronchitis, and sinusitis in humans. *C. pneumoniae* infection is quite common, and serologic studies demonstrate that 80 % of men and 70 % of women show evidence of prior infection by the age of 65 [3]. While most respiratory-acquired infections are asymptomatic or mild, it can result in pneumonia, usually involving a single lobe, and complications including life-threatening pneumonia and acute respiratory distress syndrome have been reported [4–6]. In addition, a number of human and animal studies suggest that *C. pneumoniae* infection can trigger or promote chronic inflammatory conditions, including reactive airway disease and adult-onset asthma (reviewed in [7, 8]), chronic obstructive pulmonary disease [9], and atherosclerosis (reviewed in [10–12]).

Like all Chlamydia species, *C. pneumoniae* displays a unique intracellular dimorphic lifestyle that sets it apart from other bacterial species (reviewed in [13]). There are two major developmental forms that are recognized: the infectious, but metabolically inert form, known as the elementary body (EB); and the replicative form known as the reticulate body (RB). The developmental cycle begins with cellular attachment and entry of the EB, which then rapidly converts to the RB form and begins to replicate by binary fission. These RBs grow and divide within a unique membrane-bound cellular inclusion, and at some point mid to late in the infectious process, RBs asynchronously differentiate back to EBs, and the bacterial forms are eventually released coincident with cell lysis into the extracellular space, around 40 to 72 h post infection. Then, a new round of infection and development begins with the EBs.

In addition to this productive, lytic lifestyle that releases infectious EBs, Chlamydia species have been shown *in vitro* to enter a persistent state, characterized by the development of large, irregular forms known as aberrant bodies under conditions that include IFN-γ treatment and antibiotic exposure [14–16]. Under these conditions, development stops at this aberrant form and infectious EBs fail to develop. In some cases, growth can resume when the treatment is removed, and the lytic cycle is completed. Whether this aberrant growth is relevant *in vivo* remains of great debate, but it has been hypothesized as a mechanism by which the pathogen could persist and disseminate to distant sites. For example, detection of chlamydial antigens associated with persistence *in vitro* has been reported in atherosclerotic lesions, suggesting a chronic or persistent infectious state [17, 18].

Originally considered a human-specific pathogen, the host range for *C. pneumoniae* has been expanded to include other mammals such as horses [19], koalas and other marsupials [20–22], as well as amphibians and reptiles [23–25]. Genomic studies suggest, in fact, that circulating human isolates originated from a zoonotic source [26]. Four human *C. pneumoniae* strains have undergone genomic sequencing: AR39, CWL029, J138 and TW183. As noted by Mitchell et al., the results reveal remarkable clonality of these isolates, with 99.9 % conserved gene order and organization, few deletions, and less than 300 single nucleotide polymorphisms [22], suggesting that introduction into humans is a relatively recent event in terms of evolution. Consequently, there has been little interest in completing genomic sequencing of other *C. pneumoniae* isolates, and comparative studies of the biological activity of different strains have been limited.

Our laboratory has an interest in both the acute respiratory response to *C. pneumoniae* infection as well as chronic pathogen-induced complications, such as atherosclerosis, and we have utilized both the respiratory strain AR39 as well as an atheroma isolate designated AO3 [27] for our investigations. We hypothesized that a strain associated with chronic inflammation, such as the atheroma-associated strain AO3, might benefit from inducing a less robust acute inflammatory response to the initial respiratory infection, allowing the bacteria to disseminate and persist more readily. Here we report a side-by-side comparison of AO3 and AR39, specifically looking at both *in vitro* and *in vivo* bacterial growth and host responses, to determine if these two isolates display important biological differences during respiratory infection that could predict the association with chronic complications.

## Results

### Growth of AO3 and AR39 in L929 fibroblasts and macrophages is similar

**Fig. 1:**
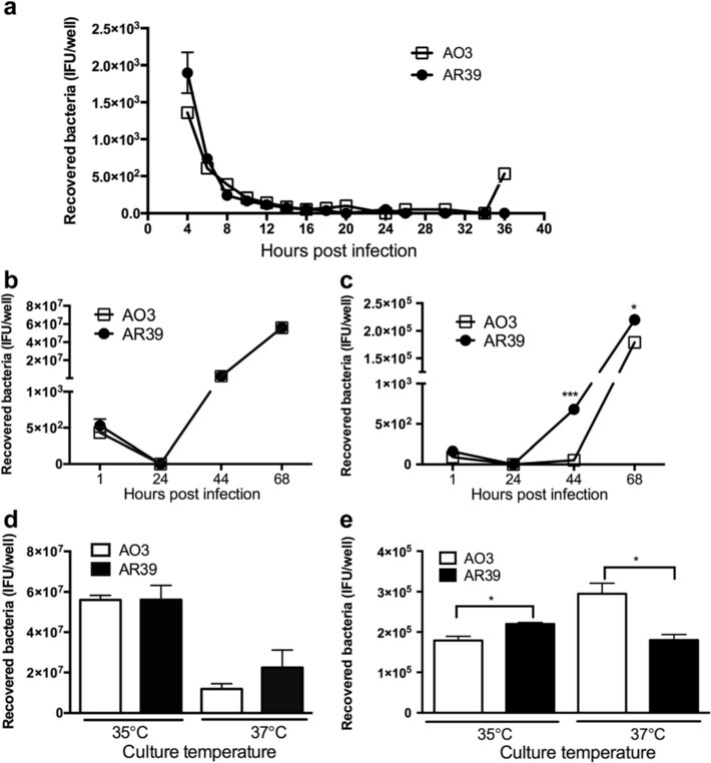
Comparative growth of *C. pneumoniae* strains AO3 and AR39. **a**-**c** Recovered AO3 and AR39 from infected L929 cells (**a**, **b**) or BMDM (**c**). Cells were incubated at 35 °C and quantitative culture was carried out at the indicated time points, as described in the Methods. **d**, **e** Quantitative culture of AO3 and AR39 infected L929 (**d**) or BMDM (**e**) grown at 35 °C vs. 37 °C growth temperature. Cells were harvested at 48 hpi for L929 and 68 hpi for BMDM, and quantitative culture was carried out as described in the Methods. For panels **a**, **b** and **d**, the inoculum was 4x10^4^ IFU (MOI 1:1). For panels **c** and **e**, the inoculum was 2x10^5^ (MOI 5:1). Significance: *, *p* ≤ 0.05; ***, *p* ≤ 0.001; compared the recovered bacteria number of AO3 with AR39 in the same time point. Data is representative of 2-3 independent experiments

*C. pneumoniae* strains AO3 and AR39 were inoculated into murine L929 fibroblasts, and cultured at the standard growth temperature of 35 °C that has been established in the literature [28]. At various time points post inoculation, the cells were lysed and the yield of infectious progeny was determined by quantitative culture. To examine the early events in primary infection, we conducted a careful titration over the initial 36 h of infection following inoculation with an identical IFU. As shown in Fig. 1a, the recovery of viable organisms rapidly decreased over the first 20 h post infection (hpi), consistent with internalization and differentiation of the EB form into the non-cultivatable RB form. By 16 hpi, no infectious progeny could be recovered for either strain. The identical titer for both strains over this time period suggests that the early events surrounding attachment, invasion, and differentiation are similar. After 24 h, we found the recovery of organisms to again be nearly identical. As shown in Fig. 1b, the titer of recovered EBs for AO3 and AR39 was identical at 44 and 68 hpi, with both strains demonstrating a significant amplification of the inoculum by more than 3 logs (4x10^4^ vs. 6x10^7^ IFU).

*C. pneumoniae* is one of the *Chlamydia spp*. that can productively infect macrophages. We next asked if growth in macrophages differed between the two strains by conducting quantitative culture in murine bone marrow derived macrophages (BMDM). Cells were infected with AO3 or AR39, and lysates were harvested and quantified again at 24, 44 and 68 hpi. In this case, we recovered AR39 but not AO3 at the earlier time point of 44 hpi (Fig. 1c), suggesting that the differentiation from RB phase back to the EB phase might be delayed in AO3 relative to AR39 within macrophages. However, both strains released infectious EBs at the later time point of 68 hpi, with the yield being higher for AR39 compared to AO3 in a small but statistically significant fashion. Titration beyond 68 hpi was not pursued as it would be confounded by a second round of infection. Of note, the yield from BMDM was largely unchanged from the original inoculum (2x10^5^ IFU), demonstrating the inefficiency of replication for both strains in macrophages.

While *C. pneumoniae* is commonly cultivated at 35 °C *in vitro*, the preferred temperature for growth of the mammalian host cells is 37 °C. We next asked if altering the growth temperature for AO3 and AR39 would differentially impact the yield of infectious EBs. Growth in both L929 fibroblasts and macrophages was repeated, this time comparing the yield when infected cells were cultured at 35 °C vs. 37 °C. For simplicity, the midpoint of log-phase growth at 35 °C, or 48 hpi, was chosen to generate comparative data in the L929 fibroblasts. Overall, the yield at 37 °C was reduced compared to bacteria grown at 35 °C in the L929 fibroblasts (Fig. 1d), and while there was a trend towards a higher yield for AR39 compared to AO3 at 37 °C, it did not reach statistical significance. Comparative growth in macrophages under the two temperatures was determined at the later time point of 68 hpi given the delayed development in this cell type. Surprisingly, we observed a difference between the two strains depending on the temperature of the cultured cells (Fig. 1e). The quantity of EBs recovered was slightly greater for AR39 compared to AO3 when cells were cultured at 35 °C; however, at 37 °C, the yield was greater for AO3. While the biological significance of such small differences is unclear, it does suggest that AO3 is more tolerant of growth at a higher temperature compared to AR39. Thus, at the host temperature of 37 °C, AO3 might have a growth advantage over AR39 in macrophage-like cells.

### Both AO3 and AR39 form plaques *in vitro*

To further assess the infectivity of the two strains, we examined the comparative plaquing efficiency of AO3 vs. AR39. To our knowledge, there have been no previous published reports on the ability of *C. pneumoniae* to form plaques *in vitro*. In fact, Gieffers and colleagues utilized a focus-forming assay to identify clonal variants of *C. pneumoniae* as the species would not form plaques using McCoy cells [29]. We first compared a number of cell lines to see if we could enhance plaquing efficiency for *C. pneumoniae* by varying the host cell, testing HeLa, Hep-2, L929, HL and McCoy cells. We found that *C. pneumoniae* was capable of forming plaques within L929 fibroblasts (data not shown), thus this cell line was used for the subsequent plaque assays described below.

**Fig. 2:**
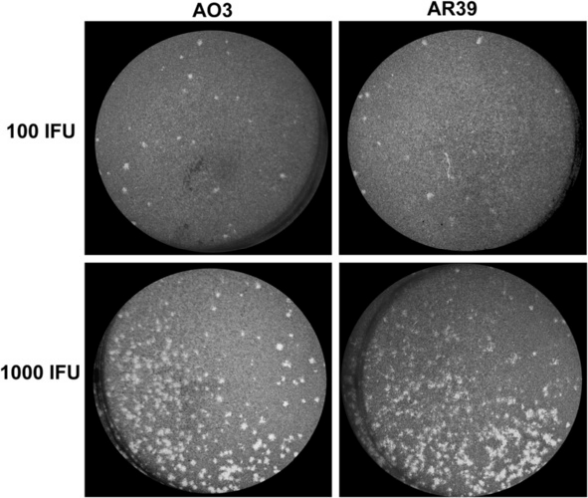
Comparison of plaques formed by *C. pneumoniae* strains AO3 and AR39. Shown are representative images of individual plaques formed following infection of L929 fibroblasts with the indicated inoculum, using the plaque assay described in the Methods section. Plaque sizes were determined to be 0.736 ± 0.033 mm for AO3 and 0.664 ± 0.035 mm for AR39 (mean ± SEM, n = 25; no significant difference). Data is representative of 3 independent experiments

To determine the efficiency of *C. pneumoniae* to form plaques, strains AO3 and AR39 were titrated in parallel using the modified plaque assay described in the Methods section. As shown in Fig. 2, we observed the formation of small plaques with both AO3 and AR39 after 15 days, in a dose-dependent manner, albeit with low efficiency. The efficiency of plating (PFU recovered/IFU inoculated) was determined to be 0.38 ± 0.09 for AO3 vs. 0.36 ± 0.13 for an inoculum of 100 IFU per well (mean ± SEM). In addition to the reduced efficiency of forming plaques, the plaque size for both AO3 and AR39 inoculated monolayers was also small (Fig. 2), with a diameter measuring 0.736 ± 0.033 mm for AO3 and 0.664 ± 0.035 mm for AR39 (mean ± SEM). Neither the efficiency of forming plaques nor the plaque size differed in a statistically significant manner between AO3 and AR39, suggesting that both strains replicate and form inclusions equally well. Thus, *C. pneumoniae* is capable of forming small plaques with reduced efficiency compared to what has been published with other species of *Chlamydia*, and we did not detect significant differences between the two strains that we tested.

### Inflammatory cytokine response to AO3 and AR39 infection differs *in vitro*

The subtle differences in bacterial growth that we observed between the two strains in BMDM suggested that the host response to the intracellular organisms might also differ. To test this, we inoculated BMDM with AO3 or AR39 at varying MOIs, and allowed the infection to proceed at either 35 °C (optimum temperature for the pathogen) or 37 °C (optimum temperature for the host). To avoid the confounding issue of cytokine feedback, supernatants were harvested at 24 hpi, and assayed for inflammatory cytokines. It should be noted that this time point is too early to identify intracellular inclusions in macrophages by routine microscopy for *C. pneumoniae* which has a much longer developmental cycle than other *Chlamydia* species. However, the titer for all stocks was confirmed as detailed in the Methods section, thus ensuring an accurate inoculum.

**Fig. 3:**
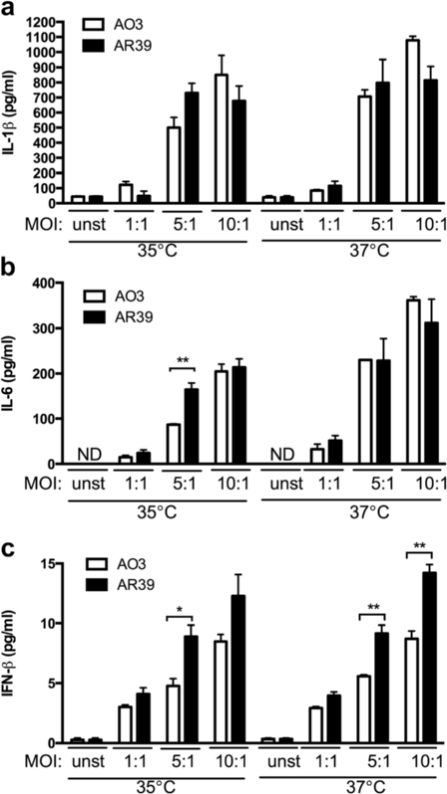
Cytokine induction in BMDM infected with *C. pneumoniae* strains AO3 or AR39. BMDMs were prepared as described in the Methods. The cells were infected with AO3 or AR39 at the indicated MOI, and incubated at 35 °C or 37 °C. Supernatant was harvested at 24 h post infection and assayed for (**a**) IL-1β, (**b**) IL-6 and (**c**) IFN-β. Graphs depict the mean ± SEM, and are representative of 3 independent experiments

We observed no difference between the two strains in terms of their ability to induce cytokine secretion for most of the mediators tested within this time frame, regardless of the incubation temperature. For example, there was no statistically significant difference between the two strains in terms of their ability to induce secretion of IL-1β (Fig. 3a). Secretion of IL-10 and TNF-α induced by AO3 and AR39 infection was also the same (data not shown), while IL-6 secretion was higher for AR39 but only for one MOI and only at 35 °C (Fig. 3b). Interestingly, although *C. pneumoniae* is known to be a weak inducer of type I IFNs [30], we found that AR39 induced significantly higher IFN-β than AO3 at both temperatures tested (Fig. 3c). Thus, AR39 appears to be more inflammatory than AO3 for a subset of cytokines, and in particular for the type I IFNs.

### AR39 induces more severe acute pulmonary disease compared to AO3 *in vivo*

**Fig. 4:**
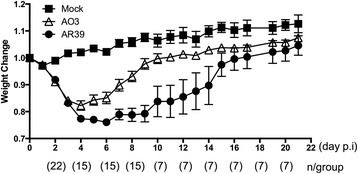
Kinetics of weight loss in mice infected with *C. pneumoniae* strains AO3 or AR39. C57BL/6 mice were intranasally infected with *C. pneumoniae* strain AO3 or AR39, or mock infected, as described in the Methods. After infection, mice were monitored and weighed daily. At predetermined time points, a subset of the mice were euthanized for analysis of histopathological changes and bacterial culture. Percent body weight was calculated compared to body weight at the time of inoculation, and is graphed on the y-axis. The number mice per group are indicated in parentheses below the x-axis. Statistical analysis was carried out as described in the Methods section. Significance: *p* < 0.05 for overall effect of condition on days 2-9 and borderline (*p* = 0.05-0.1) on days 10 and 11, as determined by ANOVA F-test (adjusted p-value). Within this significant F-test period, *p* < 0.05 days 6-9 and borderline (*p* = 0.05-0.1) on day 5 for AO3 vs. AR39. Data is representative of 2 independent experiments

The growth and development of an obligate intracellular pathogen like *C. pneumoniae* is tightly linked to the host cell. Given the differences in bacterial growth depending on the host cell type and culture temperature, combined with the trend for induction of more proinflammatory cytokines by AR39 *in vitro*, we asked whether the two strains might differ in terms of the host response to infection *in vivo*. To test this, C57BL/6 mice were infected intranasally with AO3 or AR39, or mock infected with PBS alone, and then monitored for both the clinical and pathological response over time, evaluating the mice at time points likely to reflect the acute (day 3), mid (day 9) and recovery (day 22) phases of infection [30, 31]. The overall effect of condition on the clinical finding of weight loss was found to be statistically significant on days 2 through 9, and the effect was borderline (between 0.05 and 0.10) on days 10 and 11. Compared to mock infected mice, both groups of *C. pneumoniae*-infected mice lost a significant amount of weight after inoculation (Fig. 4). For AR39 infected mice, this was statistically significant on days 2-11, while for AO3 mice it was significant on days 2-8. The greatest weight loss occurred over the first 4-6 days, after which time both groups of *C. pneumoniae* infected mice gradually regained the lost weight and there was no difference between the three groups after day 15. Pairwise analysis between AO3 vs. AR39 infected mice revealed a statistically significant difference on days 6-9, all within the period of time when the overall F-test was significant. Thus, the AR39 infected mice had more severe weight loss in response to infection compared to the AO3 infected mice.

**Fig. 5:**
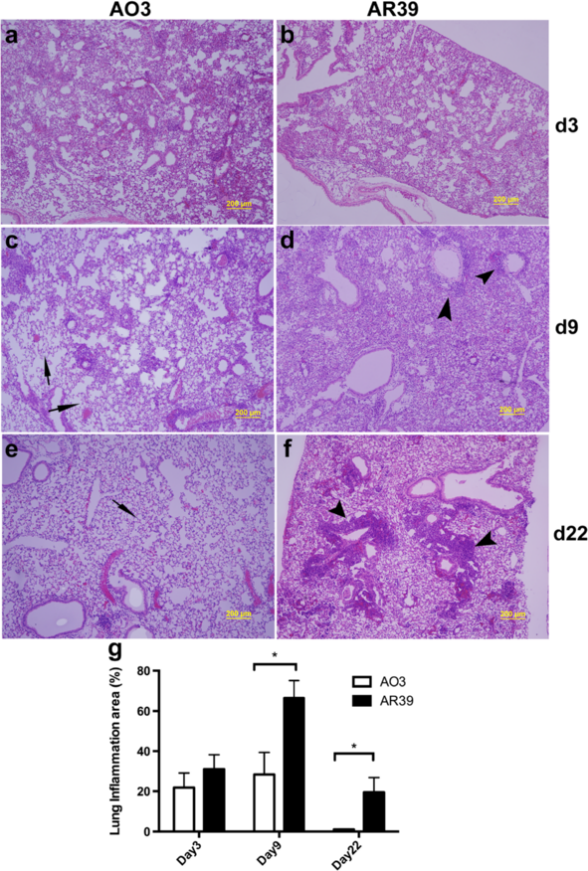
Histopathological changes in the lungs of mice infected with *C. pneumoniae* strains AO3 or AR39. C57BL/6 mice were intranasally infected with *C. pneumoniae* strain AO3 or AR39, or mock infected; at designated time points (day 3, day 9 and day 22), mice were euthanized and the lungs were processed, as described in the Methods section. **a**-**f** Representative images of H&E stained lung tissue from 1 μm resin-embedded sections; n = 3 mice per group. Arrow heads identify areas of inflammation while the arrows identify normal lung. **g** Quantified inflammation area from lung sections was generated using Image-J software analyzing both lungs, *n* = 3 mice per group. Significance: *, *p* ≤ 0.05. Data is representative of 2 independent experiments

When we looked at the local inflammatory response in the lungs, AR39 infected mice displayed significantly more inflammation in the lungs, as determined by H&E staining, compared to AO3 infected mice (Fig. 5a-f). At day 9, the areas of inflammation were more extensive in the AR39 infected mice compared to the AO3 infected mice, with little normal appearing lung tissue visible. At day 22, while the inflammation in the AO3 infected mice had completely resolved, patchy inflammation was still apparent in the AR39 infected mice. These differences were quantifiable and statistically significant (Fig. 5g). Thus, mice infected with the respiratory isolate, AR39, developed more severe acute lung disease than mice infected with the atheroma isolate AO3.

**Fig. 6:**
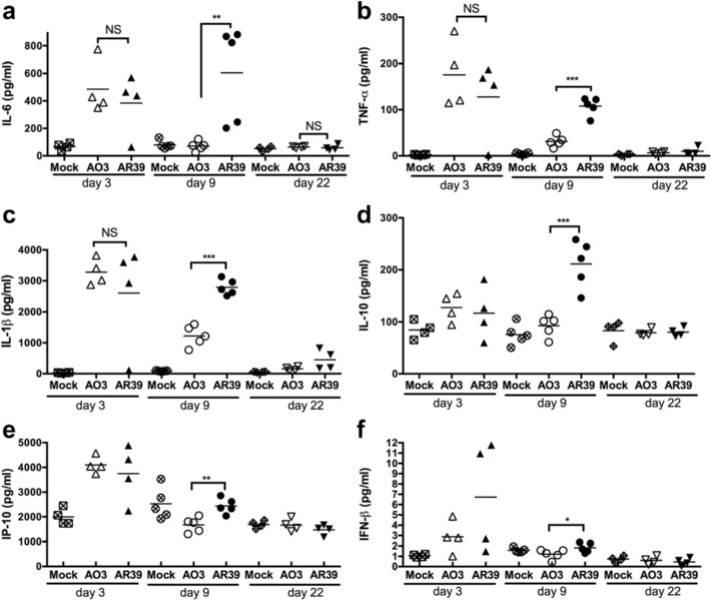
Cytokine induction in the lungs of AO3 or AR39 infected mice. Group mice were infected with AO3 or AR39, or mock infected, and euthanized at designated time points as described in figure four and the Methods section. Removed lung were homogenized and the lung homogenates were assayed for cytokines IL-6 (**a**), TNF-α (**b**), IL-1β (**c**), IL-10 (**d**), IP-10 (**e**) and IFN-β (**f**) by ELISA. Each data point represents one mouse. Horizontal bar represents the mean. Significance: NS, not significant; *, *p* ≤ 0.05; **, *p* ≤ 0.01; ***, *p* ≤ 0.001. Data is representative of 2 independent experiments

Consistent with the apparent histological differences in inflammation, AR39 infected mice also had higher levels of a number of cytokines in the lung tissue at day 9, including IL-6, TNF-α, IL-1β and IL-10; the IRF-regulated cytokines IP-10 and IFN-β were also higher in the lungs of AR39 infected mice (Fig. 6). By day 22, however, similar to the improvement in weight loss and lung inflammation, cytokine levels returned to normal in both groups of infected mice.

**Fig. 7:**
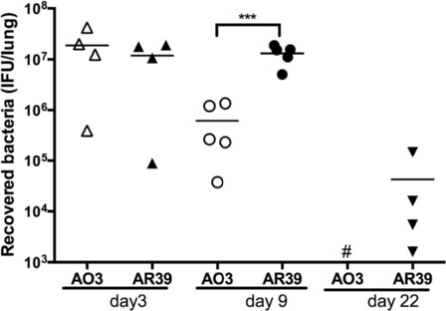
Bacterial load in the lungs of AO3 or AR39 infected mice. Lung homogenates from AO3 or AR39 infected mice were performed *C. pneumoniae* quantitative culture as described in the Methods. Each data point represents one mouse and the horizontal bar represents the mean. Significance: NS, no significant difference; ***, *p* ≤ 0.001. Data is representative of 2 independent experiments

Finally, recovery of bacteria from lung tissue was also higher for AR39 at day 9 compared to AO3, and even out to day 22 there were still mice from which we could recover AR39 from lung tissue (Fig. 7). However, we found no significant difference in the ability of the bacteria to disseminate to the spleen (Table 1). While there was a trend for more frequent recovery of bacteria at this distant site in the AR39 infected group, it did not reach statistical significance, likely reflecting the overall low frequency of dissemination of live (cultivatable) bacteria in both groups.

**Table 1:** Number of mice with positive *C. pneumoniae *culture from spleen

Strain	Day 3 (n = 7)	Day 8 (n = 8)	Day 22 (n = 7)
AO3	1	2	1
AR39	2	5	2

*p*-value not significant for AO3 vs. AR39 at any time point

## Discussion

This is the first comparison of the growth dynamics of *C. pneumoniae* strains AO3 and AR39 *in vitro* and *in vivo*, and the first demonstration that strain differences can impact the host response to infection using a mouse model. Both AO3 and AR39 displayed nearly identical growth patterns *in vitro*, and both strains were capable of forming plaques under our culture conditions. This latter observation is relevant to the *Chlamydia* field as previously published data suggested that *C. pneumoniae* could not be cloned using the traditional plaque assay. However, subtle differences between the strains in terms of growth and development were observed depending on the cell type and growth temperature. Both strains grew better when cultured at 35 °C compared to 37 °C, but only in L929 fibroblasts. While both strains could infect macrophages, AR39 had the growth advantage at 35 °C while AO3 had the growth advantage at 37 °C, at least *in vitro*. The explanation for why AO3 has the growth advantage at 37 °C is unclear. Data from Bonanomi suggests that at 37 °C, AR39 has a longer lag phase than AO3, although comparisons were not made to growth at 35 °C in that study [32]. While AO3 had a slight growth advantage at 37 °C *in vitro* in our hands, the fact that we recovered more AR39 than AO3 in the lungs of infected mice would suggest that *in vivo* infectivity, growth and development might not necessary parallel what is observed *in vitro*.

In addition to differences in bacterial growth and development, we also observed important differences in terms of the host response to the two different strains. The induction of type I IFNs, and to a lesser extent IL-6, from infected BMDM *in vitro* was greater in AR39 infected cells compared to AO3. This occurred in spite of the fact that we observed a greater yield of AO3 from BMDM cultured at 37 °C compared to cells infected with an identical inoculum of AR39. Thus, we cannot explain this difference in cytokine induction simply from a larger bacterial burden. The more compelling data, however, demonstrates that mice experienced more severe weight loss and more severe lung inflammation when infected via the intranasal route with AR39 compared to AO3. This is consistent with our recently published data correlating the induction of type I IFNs *in vitro* with disease severity *in vivo*, using AR39 in two mouse strains [30]. In this study, we observed that impaired bacterial growth predicted impaired host tolerance of infection *in vitro* and *in vivo* through the activation of type I IFN signaling pathways. Thus, the growth differences of AR39 and AO3 in macrophages at 37 °C could similarly lead to their varying ability to activate the type I IFN signaling pathways, and the subsequent differences in the host response. Taken together, these data demonstrate that the type I IFN response is a key factor in determining the ability of the host to tolerate *C. pneumoniae* infection, and it can be regulated by either host or pathogen characteristics.

At this time, we can only speculate as to the basis for the differences in bacterial growth and the host response to infection that we observed. There was no apparent difference in infectivity or growth *in vitro* between these two strains, and both were capable of forming plaques in cell culture. While we do not have the genome sequence for AO3 to compare with the published sequence of AR39, as noted earlier, there is remarkable clonality between all the sequenced isolates of *C. pneumoniae*, with 99.9 % conserved gene order and organization, and few deletions and polymorphisms [22]. However, small differences at the level of the genome could lead to significant differences in gene regulation that might account for the differences in growth, the timing of expression of specific pathogen-associated inflammatory ligands, and eventually the differences in the host response. Molestina et al. reported that the *omp1* gene from AO3 displays 5 nucleotide changes resulting in nonsynonymous codons compared to the respiratory strains TW183 and AR39 [33]. Like us, they also observed that AO3 displayed the lowest stimulatory activity in terms of upregulation of proinflammatory cytokines and adhesion molecules in human endothelial cells compared to the respiratory strains AR39 and TW183. This latter point is reassuring as it suggests that our observation is more likely to be driven by the specific strain types rather than phenotypic drift of clinical isolates passaged *in vitro*.

## Conclusions

In summary, our data suggests that, despite the limited genetic variability among *C. pneumoniae* strains, phenotypic differences exist between human isolates that lead to differences in inflammatory potential. The result is variability between strains in terms of the clinical course of pneumonia, and potentially could impact associated diseases, such as atherosclerosis and asthma. The basis for the differences that we observed between these two clinical strains is not clear at this time, and would require more extensive genetic and transcriptional profiling that is beyond the scope of this report. In the context of our previous study [30], our data is consistent with an important role for activation of type I IFNs in driving many aspects of *C. pneumoniae* virulence, and demonstrates that both host and pathogen factors can contribute to outcome. The ability to identify specific bacterial traits that can predict the development of complications would greatly benefit the field.

## Methods

### Ethics statement

All animal use protocols were approved by the Institutional Animal Care and Use Committee (IACUC) of Boston University, in accordance with the recommendations in the Guide for the Care and Use of Laboratory Animals of the National Institutes of Health. Every effort was made to minimize discomfort, pain and distress in the animals. Boston University is accredited by the Association for Assessment and Accreditation of Laboratory Animal Care (AAALAC).

### Reagents

RPMI1640 was purchased from BioWhittaker® (Lonza Walkersville, MD, USA). Fetal Bovine Serum (FBS; low endotoxin) was purchased from Hyclone (Logan, Utah). Modified Eagles Medium (2x, no phenol red) was purchased from Gibco Life technologies. Plaque Assay Agarose was purchased from BD Biosciences (San Jose, CA). Renografin-60 was purchased from Bracco Diagnostics Inc. (Princeton, NJ).

### Propagation of chlamydiae

*Chlamydia pneumoniae* strain AO3 was kindly provided by Dr. Charlotte Gaydos (Johns Hopkins University, Baltimore, MD) and strain AR39 was obtained from Dr. Li Shen (Louisiana State University, New Orleans, LA). Gradient purified AO3 and AR39 were prepared after propagation in L929 fibroblasts, as described previously [31], and the titer calculated as inclusion forming units (IFU) per ml. To ensure accurate titers, all aliquotted stocks were frozen at -80 °C, and thawed once, at the time of use. All stocks used for these studies tested negative for *Mycoplasma* contamination by PCR [34].

### Plaque assay

The protocol for the plaque assay of *Chlamydia pneumoniae* was a variation on previously published protocols [35]. Briefly, L929 cells were plated in 6-well dishes at a density of 10^6^ cells per well, inoculated with *C. pneumoniae* AO3 or AR39 in 10-fold serial dilutions, and the plates were centrifuged at 1500 x g at 35 °C to initiate infection. After centrifugation, the plates were incubated for 30 min at 35 °C/5 % CO_2_, after which the medium was replaced by an overlay of plaque assay mixture prepared by mixing 2 % agarose (42 °C) with an equal volume of 2x MEM culture medium (37 °C) containing 20 % FBS, cycloheximide (2 mg/ml), non-essential amino acids (2x), HEPES (2x), glucose (0.8 %) and gentamicin. The plates were incubated at 35 °C/5 % CO_2_ for 14-15 days. Additional plaque assay mixture was added every four days. The plaques were visualized by staining live cells with 0.3 % neutral red. The efficiency of plating (EOP) was calculated by dividing plaque forming units (PFU) formed under each condition by the IFU inoculated. Data shown was pooled from 3 independent experiments.

### Mice

C57BL/6 J mice used in this study were purchased from Jackson Laboratory (Bar Harbor, ME). All animals were housed in groups of 3-5 mice per cage in a controlled environment (temperature 20-22 °C, 12:12 h light:dark cycle), given free access to food and water, and maintained under the supervision of veterinary staff from the Laboratory Animal Science Center (LASC) at Boston University Medical Center.

### Preparation of bone marrow-derived macrophages

Bone marrow derived macrophages (BMDM) were prepared as described previously [31]. Briefly, bone marrows were flushed from femurs and tibiae of C57BL/6 mice, 6-8 weeks age, and the cells were cultured in RPMI 1640 supplemented with 10 % FBS, 20 μg/ml of gentamicin, and 20-30 % (v/v) of L929 condition medium (containing M-CSF). The cells were incubated at 37 °C/5 % CO_2_ for 7-9 days to allow differentiation of macrophages; gentamicin was removed from the culture medium one day prior to infection with chlamydia. Infection was initiated, using the indicated MOI, by centrifugation at 1500 x g for 1 h at 35 °C, and infected cells were then incubated at 35 °C or 37 °C in a 5 % CO_2_ environment for 24 h.

### Murine intranasal infection model

Groups of 15 (mock), 22 (AO3) and 22 (AR39) C57BL/6 mice, 7 to 8 weeks of age, were inoculated with *C. pneumoniae* strain AO3, AR39 or mock via the intranasal route under light anesthesia using ketamine/xylazine mix (60-100/5-10 mg/kg i.p.). All experimental groups were gender and age matched. Infected mice received 5 × 10^6^ IFU gradient purified AO3 or AR39 diluted in 20 μL of PBS followed by 20 μl of PBS; mock infected mice received 20 μl of PBS followed by an additional 20 μl of PBS. Mice were weighed daily, and observed for signs of distress. At days 3, 9 or 22, mice were euthanized by CO_2_ inhalation. Following collection of blood by cardiac puncture as a secondary means of euthanasia, the lungs and spleens were removed for cytokines and bacterial quantification. Lungs and spleens were homogenized in PBS using a Medimachine System (BD Biosciences, San Jose, CA). Three mice from each group were designated for histopathology and immunohistochemistry and processed as follows: lungs were inflated with 10 % neutral formalin via the trachea, removed right lungs were further fixed in formalin for paraffin embedding; left lungs were further fixed in 4 % paraformaldehyde for JB4 resin embedding.

### Quantitative culture of *C. pneumoniae*

Quantitative culture of *C. pneumoniae* in lung homogenates, spleen samples, or cell lysates was carried out as described previously [31]. Briefly, for infected tissues, serially diluted homogenized samples were inoculated in duplicate onto L929 fibroblasts seeded in a 96-well plate. Infection was initiated by centrifugation at 1500 x g for 1 h at 35 °C, and infected cells were then incubated at 35 °C/5 % CO_2_. After incubation for 48 h, a time point prior to completion of the developmental cycle and initiation of a secondary round of infection, the cells were fixed in ice-cold methanol and stained for fluorescence microscopy. Inclusions were visualized using a Chlamydia-specific LPS monoclonal antibody (gift of Dr. You-Xun Zhang, Boston Medical Center), followed by FITC-conjugated secondary antibody; cells were counter stained with Evans blue. The inclusions were counted under fluorescence microscopy to determine the chlamydial IFU per mL of sample, which was then used to calculate the total IFU per mouse. For quantitative culture of *in vitro* infected cells, L929 or BMDM were disrupted, and the supernatants were serially diluted and inoculated in duplicate onto L929 fibroblasts seeded in a 96-well plate. Inclusions were counted as described above to determine the Chlamydia concentration. The lower level of detection in this assay was determined to be 200 IFU per lung for the *in vivo* samples and 5 IFU per well for the *in vitro* samples.

### Histopathology

Embedded lung blocks were cut completely in 2.5 μm sections for resin embedded tissue and 7-8 μm sections for paraffin embedded tissue; every 10th section was stained using routine hematoxylin and eosin (H&E) as described previously [36]. Pathological changes were quantified using Image-J software [37].

### Cytokine assay

Supernatants or lung homogenates were assayed for cytokines using commercially available ELISA kits according to the manufacturer’s instructions. ELISA kits for mouse IL-6, IL-1β and IL-10 cytokines were purchased from R&D Systems (Minneapolis, MN, USA); TNF-α ELISA kits were from eBioscience. Mouse IFN-β ELISA was carried out as previously described [30] using rat anti-mouse IFN-β monoclonal Ab (I7662-10A as the capture antibody (US Biological, Swampscott, MA, USA); rabbit anti-mouse IFN-β polyclonal Ab (32400-1) as detection antibody, and mouse rIFN-β standard (12400-1), (both purchased from PBL Biomedical Laboratories, Piscataway, NJ, USA); and HRP-conjugated donkey anti-rabbit IgG (711-036-152 (Jackson ImmunoResearch Laboratories, West Grove, PA, USA). Plates were read in an ELx800 Universal Microplate Reader (Bio-Tek, Instruments, Inc., Winooski, VT, USA), and data was analyzed using SoftMax Pro 4.6 software. Each experimental condition for the *in vitro* studies was performed in triplicate.

### Statistical analysis

Data is presented as the mean ± SEM. For the comparative analysis of weight loss over time, multiple testing analyses were carried out across the 21 days. Raw p-values were generated for each day using the GLM procedure and the adjustment for multiple testing was done using the Hochberg procedure [38] in the MULTTEST procedure in SAS version 9.3. Statistically significant pairwise differences are reported only between conditions when the F-test for an overall effect is significant. Pairwise differences between quantitative culture, cytokine levels, and plaque sizes were determined using the Student’s *t*-test. Values of *p* < 0.05 were considered significant.

## Abbreviations

BMDM: Bone marrow derived macrophages
EB: Elementary body
hpi: Hours post-infection
IFN: Interferon
IL: Interleukin
IFU: Inclusion forming unit
IP-10: Interferon gamma induced protein 10
M-CSF: Macrophage colony stimulating factor
MOI: Multiplicity of infection
NS: No significance
PFU: Plaque forming unit
RB: Reticulate body
SEM: Standard error of the mean
TNF: Tumor necrosis factor.
